# Supplementary material for: Ice-volume-forced erosion of the Chinese Loess Plateau global Quaternary stratotype site
Source: Nat Commun. 2018 Mar 7;9:983. doi: 10.1038/s41467-018-03329-2 (PMC5841279; doi:10.1038/s41467-018-03329-2)
Supplement: Supplementary file 1 — Supplementary Information [file 41467_2018_3329_MOESM1_ESM.pdf]

Supplementary Information

Ice-volume-forced erosion of the Chinese Loess Plateau global Quaternary stratotype site

by Stevens et al.

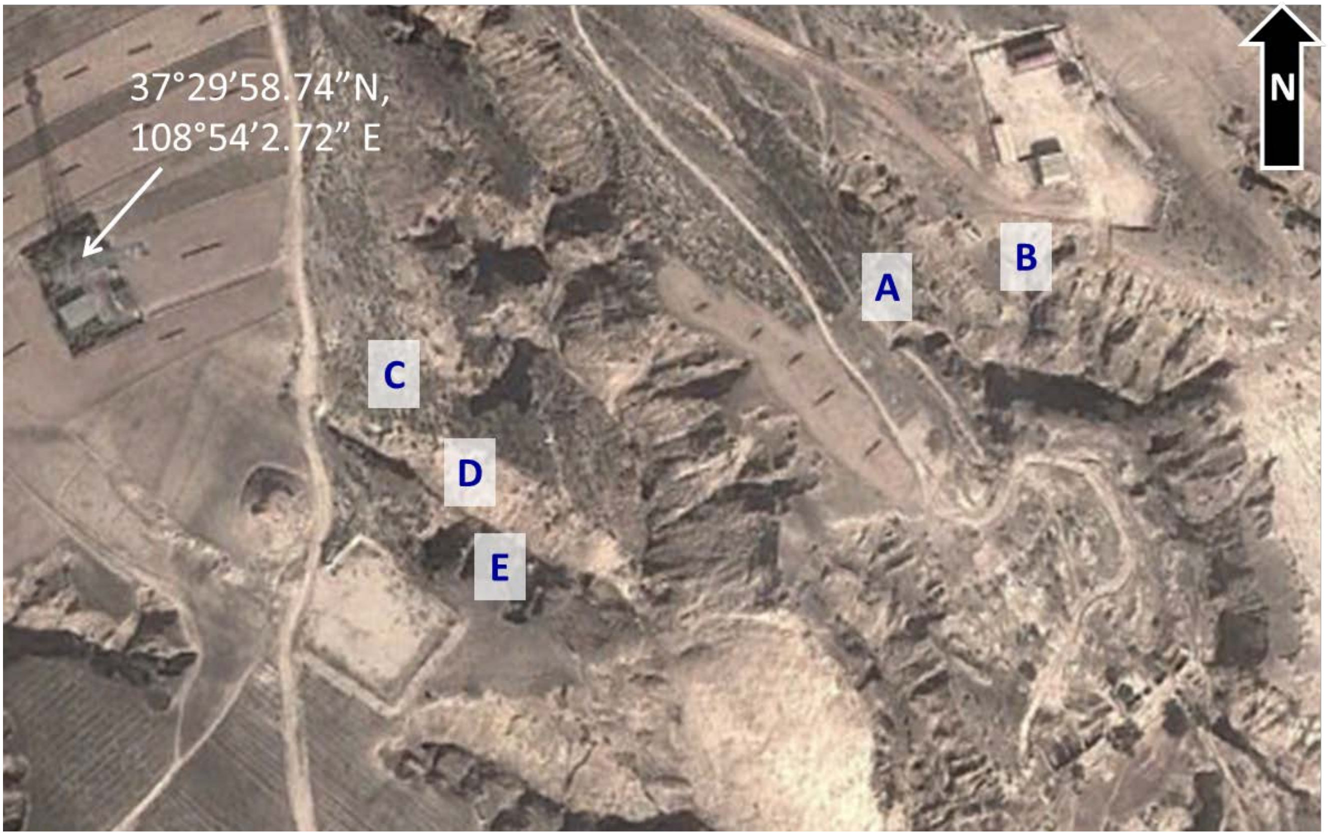

Supplementary Figure 1. Map showing the location of the different sections at Jingbian.
The distance between sections A, B and sections C, D, E is ~200 m. Map data: Google, DigitalGlobe.

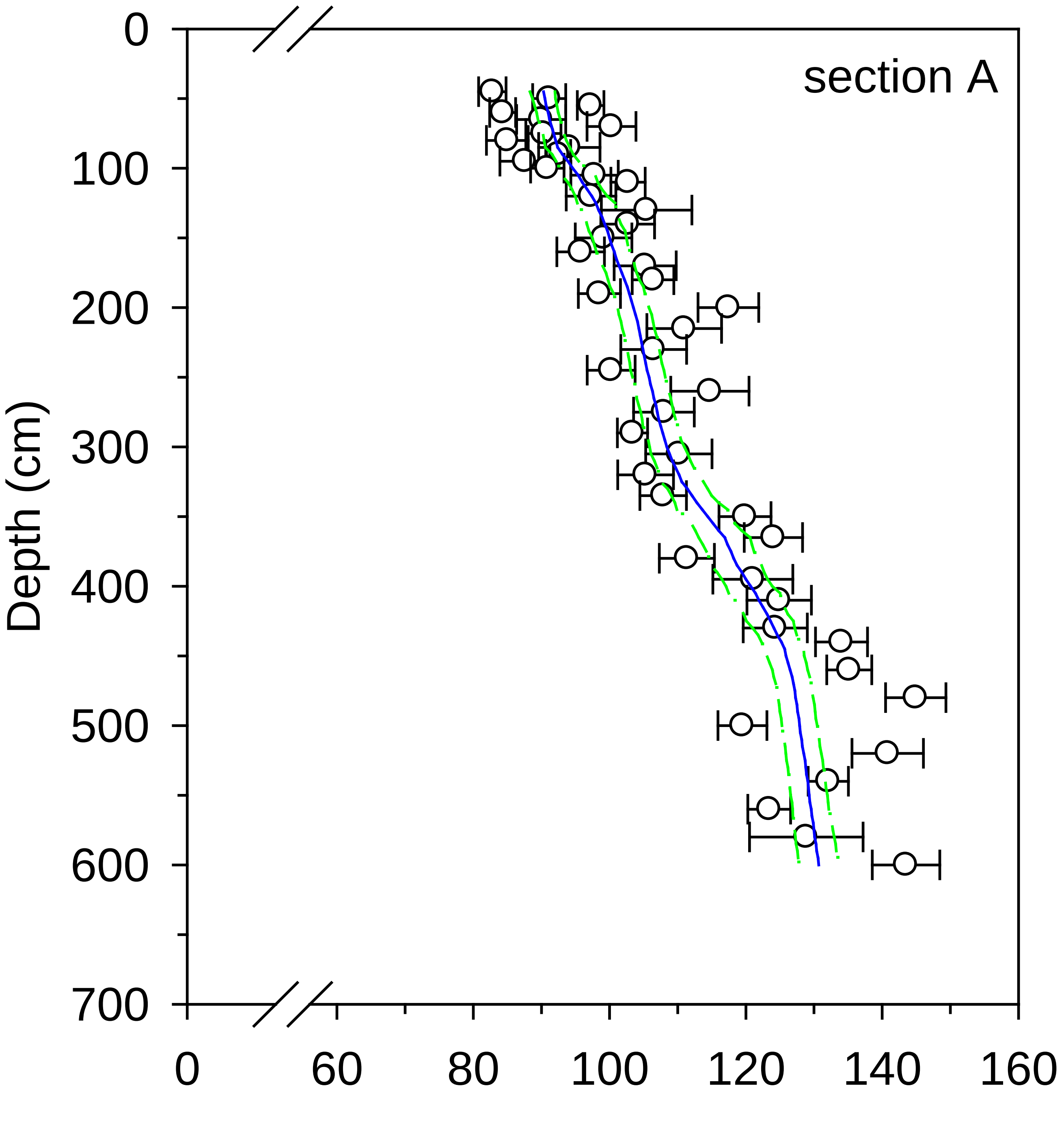

Supplementary Figure 2a. Age-depth plot for section A with Bayesian modelling shown in solid blue (weighted mean) and dashed green (min and max age) lines. Errors bars represent 1 s.d.

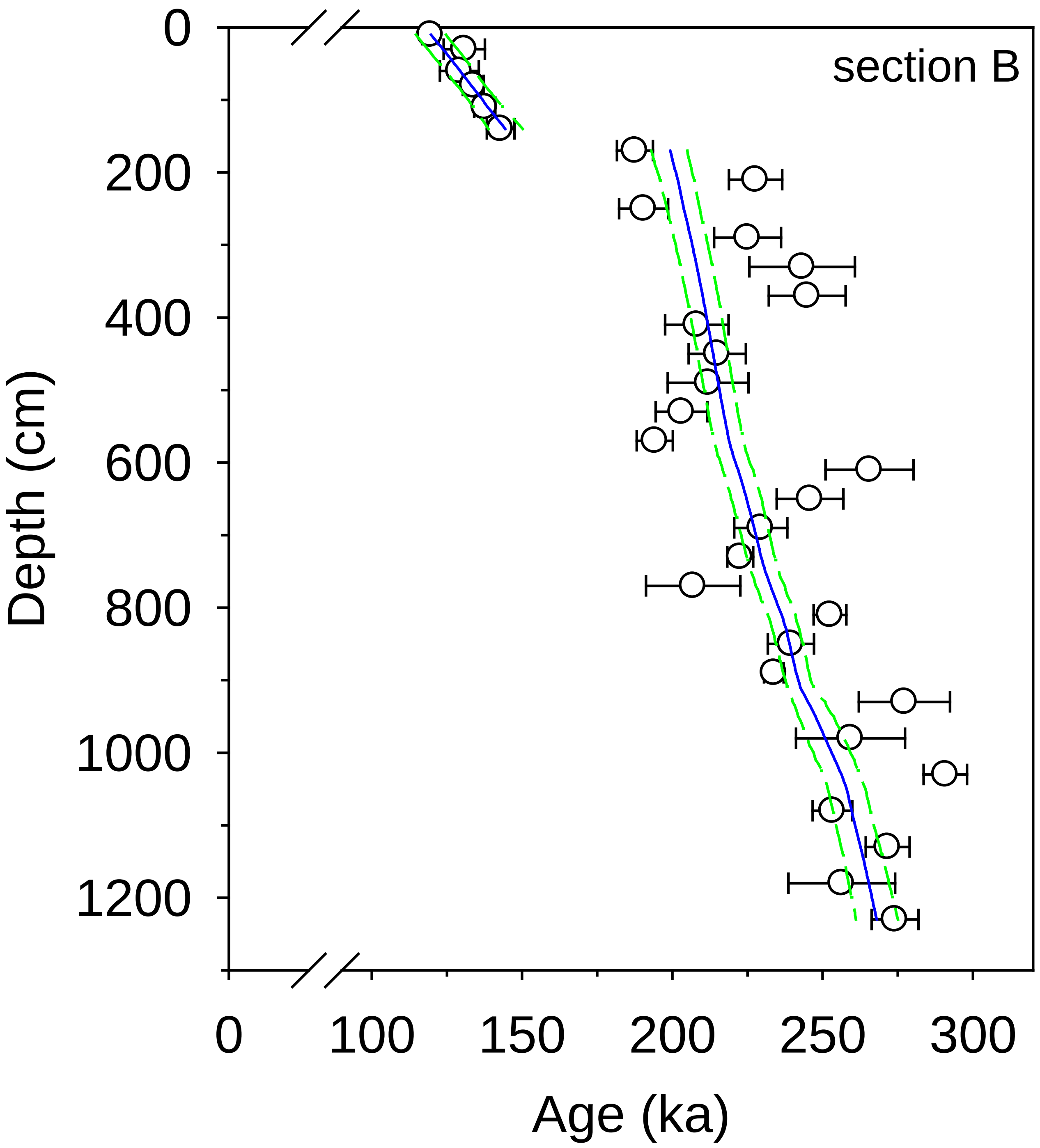

Supplementary Figure 2b. Age-depth plot for section B with Bayesian modelling shown in solid blue (weighted mean) and dashed green (min and max age) lines. Errors bars represent 1 s.d.

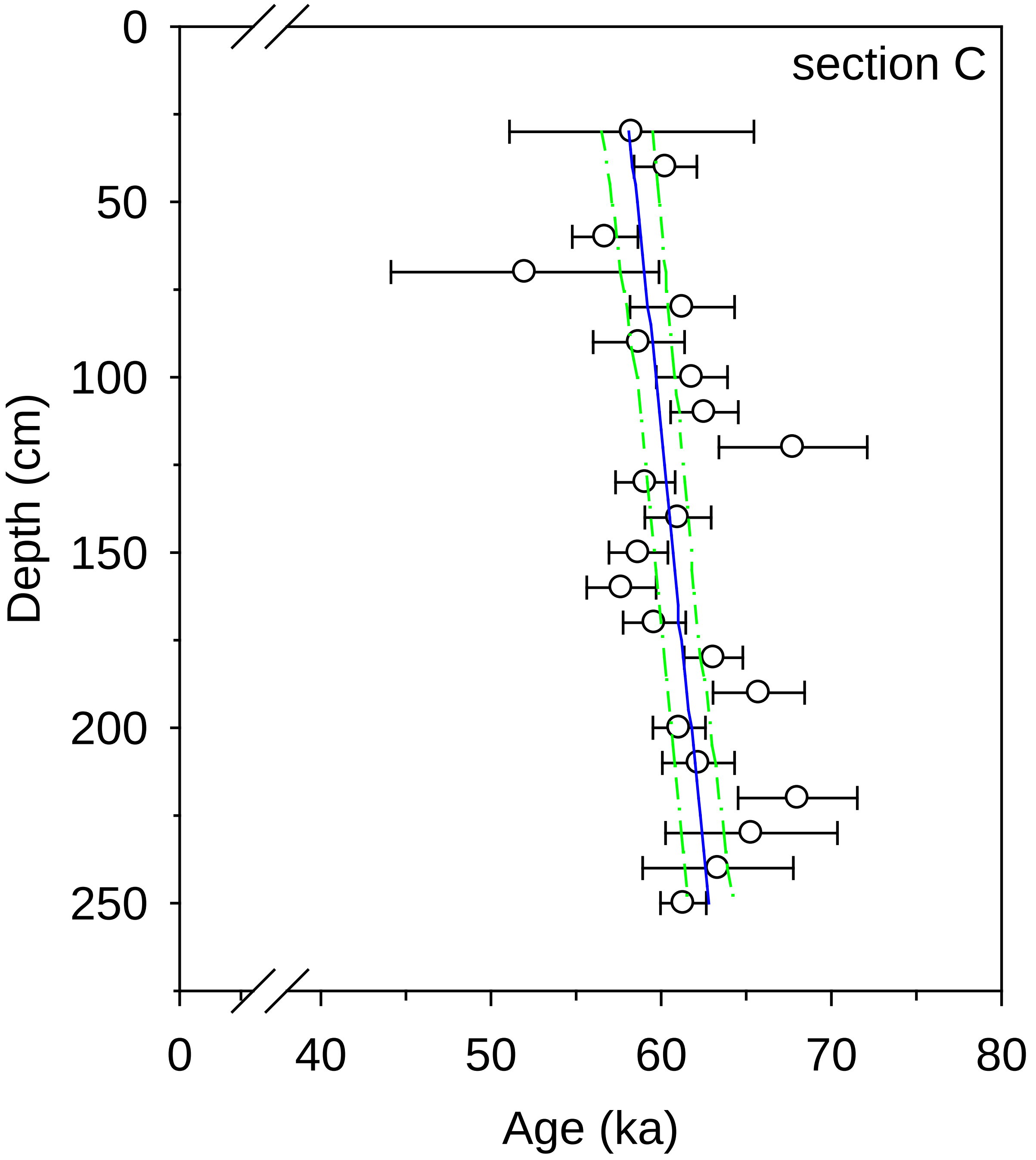

Supplementary Figure 2c. Age-depth plot for section C with Bayesian modelling shown in solid blue (weighted mean) and dashed green (min and max age) lines. Errors bars represent 1 s.d.

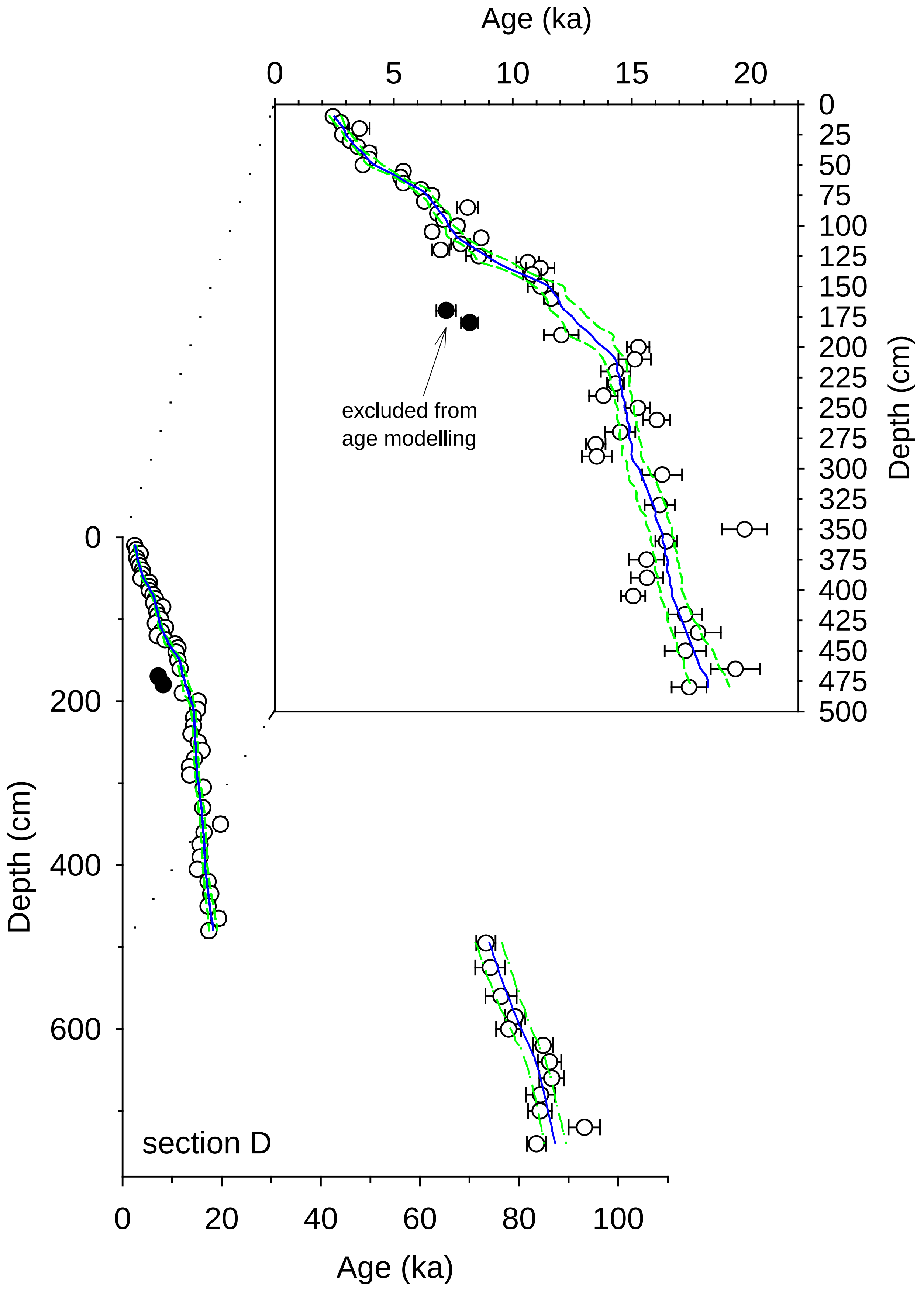

Supplementary Figure 2d. Age-depth plot for section D with Bayesian modelling shown in solid blue (weighted mean) and dashed green (min and max age) lines. Errors bars represent 1 s.d.

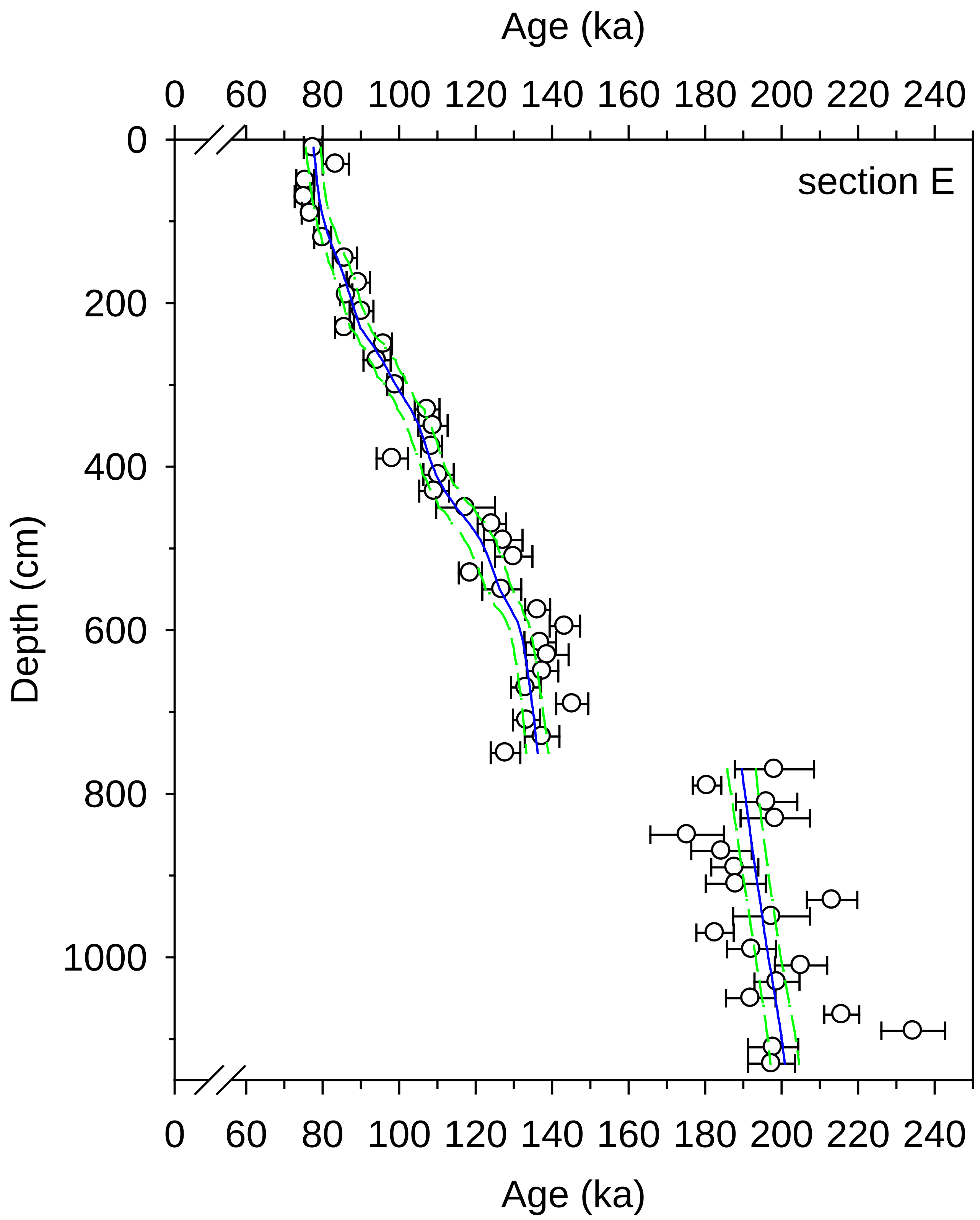

Supplementary Figure 2e. Age-depth plot for section E with Bayesian modelling shown in solid blue (weighted mean) and dashed green (min and max age) lines. Errors bars represent 1 s.d.

Supplementary Table 1. a) SAR protocol for quartz OSL; b) SAR protocol for feldspar post-IR IRSL

Step	a) Quartz SAR OSL protocol	Observed	b) Feldspar pIRIR$_{290}$ protocol	Observed
1	Dose		Dose	
2	Preheat (160-300°C, 10 s)		Preheat (320°C, 60 s)	
3	OSL (125°C , 40 s)	L_X	IRSL (T°C , 200 s)	
4	Test dose		IRSL (290°C, 200 s)	L_X
5	Cut-heat (160-300°C)		Test dose	
6	OSL (125°C , 40s)	T_X	Preheat (320°C, 60 s)	
7	Clean-out (40 s blue light at 280°C)		IRSL (T°C, 200 s)	
8	Return to step 1		IRSL (290°C, 200 s)	T_X
9			IRSL (325°C, 200 s)	
10			Return to step 1	

Note: Dose in step 1 is 0 Gy for the D_e measurement. For quartz D_e and dose recovery measurements a preheat of 260°C and a cut-heat 220°C was used. For K-rich feldspar D_e and dose recovery measurements the first IR stimulation temperature (steps 3 and 7) was kept at 200°C. The test dose in protocol b) varied between ~30 and ~70% of the dose that was measured.

Supplementary Table 2. Calculated lags between magnetic susceptibility and insolation curves

Insolation inflection point (ka)	Section	Magn. sus. inflection point (ka)	Calculated lag (ka)
245.8	B	242	3.8
201.6	E	198.3	3.3
131.2	E	125.2	6.0
131.2	A	126.4	4.8
108.8	E	102.7	6.1
108.8	A	103.6	5.2
87.4	E	78.5	8.9
15.4	D	83.5	3.9
			**Average (n=9) = 4.9**
			**s.d. = 2**
