# Supplementary material for: Ice-volume-forced erosion of the Chinese Loess Plateau global Quaternary stratotype site
Source: Nat Commun. 2018 Mar 7;9:983. doi: 10.1038/s41467-018-03329-2 (PMC5841279; doi:10.1038/s41467-018-03329-2)
Supplement: Supplementary file 3 — Description of Additional Supplementary Files [file 41467_2018_3329_MOESM3_ESM.docx]

**Description of Additional Supplementary File**

**File Name**: Supplementary Data 1

**Description:** Excel spreadsheet containing analytical data for proxy records (stratigraphy, sand content, low-field magnetic susceptibility, bulk density), luminescence dating (quartz/feldspar age, equivalent dose, total dose rates, assumed water content, radionuclide concentrations) and output of Bayesian modelling at 5 cm depth resolution for each section.
